# Adherence to Mediterranean Diet, Physical Activity and Survival after Prostate Cancer Diagnosis

**DOI:** 10.3390/nu13010243

**Published:** 2021-01-16

**Authors:** Matteo Di Maso, Livia S. A. Augustin, Federica Toffolutti, Carmen Stocco, Luigino Dal Maso, David J. A. Jenkins, Neil E. Fleshner, Diego Serraino, Jerry Polesel

**Affiliations:** 1 Department of Clinical Sciences and Community Health, Branch of Medical Statistics, Biometry and Epidemiology “G.A. Maccacaro”, Università degli Studi di Milano, via A. Vanzetti 5, 20133 Milan, Italy; matteo.dimaso@unimi.it; 2 Epidemiology and Biostatistics Unit, Istituto Nazionale Tumori–IRCCS–“Fondazione G. Pascale”, via M. Semmola 1, 80131 Naples, Italy; l.augustin@istitutotumori.na.it; 3 Unit of Cancer Epidemiology, Centro di Riferimento Oncologico di Aviano IRCCS, via F. Gallini 2, 33081 Aviano, Italy; federica.toffolutti@cro.it (F.T.); dalmaso@cro.it (L.D.M.); serrainod@cro.it (D.S.); 4 Venetian Cancer Registry, Veneto Region, via J. Avanzo 35, 35131 Padua, Italy; carmen.stocco@azero.veneto.it; 5 Departments of Nutritional Science and Medicine, Temerty Faculty of Medicine, University of Toronto, Toronto, ON M5S 1A8, Canada; david.jenkins@utoronto.ca; 6 Clinical Nutrition and Risk Factor Modification Centre, St. Michael’s Hospital, Toronto, ON M5C 2T2, Canada; 7 Division of Endocrinology and Metabolism, Department of Medicine, St. Michael’s Hospital, Toronto, ON M5C 2T2, Canada; 8 Li Ka Shing Knowledge Institute, St. Michael’s Hospital, Toronto, ON M5C 2T2, Canada; 9 Division of Urology, Department of Surgical Oncology, Princess Margaret Cancer Center, University Health Network, Toronto, ON M5G 2C1, Canada; neil.fleshner@utoronto.ca

**Keywords:** Mediterranean diet, physical activity, prostate cancer, survival

## Abstract

Despite the considerable number of studies investigating the Mediterranean diet in prostate cancer (PCa) etiology, very few focused on cancer survival. We assessed the pre-diagnostic diet and physical activity in a cohort of 777 men with PCa diagnosed between 1995 and 2002 in north-eastern Italy; adherence to the Mediterranean diet was evaluated through the Mediterranean Diet Score (MDS). Hazard ratios (HR) of death with confidence intervals (CI) were estimated using the Cox model, adjusting for potential confounders. During 10 years of follow-up, 208 patients (26.8%) died, 75 (9.7%) due to PCa. Patients reporting MDS ≥ 5 showed a higher overall survival than those with MDS < 5 (HR = 0.74; 95% CI: 0.56–0.99). Although high physical activity was not significantly associated with overall survival (HR = 0.79; 95% CI: 0.59–1.07), the HR for all-cause death was the lowest (HR = 0.58; 95% CI: 0.38–0.90) for men reporting MDS ≥ 5 and high physical activity compared to those reporting MDS < 5 and low/moderate physical activity. No association emerged for PCa specific survival. Study findings support the beneficial impact of pre-diagnostic adherence to the Mediterranean diet and physical activity on overall survival; they are mainly driven by risk reduction in non-prostate cancer mortality, which however accounts for about 80% of death in men with PCa.

## 1. Introduction

In Europe, prostate cancer (PCa) is the most common neoplasm among men (approximately 473,000 new cases/year) and it is the leading cause of cancer deaths (approximately 108,000 deaths/year) [1]. The widespread use of prostate-specific antigen (PSA) testing, which started in Italy in the early 1990s [2], has increased the detection of latent, early-stage, and slow-growing tumors, contributing to the increasing overall survival in patients with PCa. Therefore, over 560,000 men living after a diagnosis of PCa are estimated in Italy in 2020 [3]. Cardiovascular disease is the most frequent cause of death among patients with a PCa diagnosis, especially in those with low-risk PCa [4]. Therefore, the identification of modifiable lifestyle factors affecting the long-term PCa prognosis is of great relevance.

Adherence to the Mediterranean diet has been consistently associated with reduced all-cause mortality in the general population, with similar associations across geographic areas [5]. However, life expectancy is dramatically reduced after cancer diagnosis; therefore, it is important to evaluate if a similar beneficial effect of adherence to the Mediterranean diet also applies to people with cancer. Despite the considerable number of studies investigating the Mediterranean diet in cancer etiology, only the Health Professionals Follow-up study [6] focused on survival, reporting a 22% reduction in risk of all-cause death in men with PCa who were highly adherent to the Mediterranean diet.

Physical activity has consistently been associated with lower overall mortality in men with PCa [7,8,9], as well as with lower PCa specific mortality [7,8,9,10]. Furthermore, physical activity may interact with dietary habits and potentially affect PCa prognosis. A recent study in cancer patients from the Third National Health and Nutrition Examination Survey (NHANES III) reported that a higher Healthy Eating Index and being physical active was associated with better survival [11]. Therefore, the present study aimed at investigating whether pre-diagnostic adherence to the Mediterranean Diet and physical activity were associated with PCa survival and whether an interaction between the two factors may exist.

## 2. Materials and Methods

This study analysed data from a retrospective cohort of men with PCa initially enrolled as cases in an Italian case-control study on the association between lifestyle factors and PCa risk [12]. Cohort participants were 780 consecutive patients aged 46–74 years (median age: 66 years) with incident, histologically confirmed PCa diagnosed in the period 1995–2002, resident in Friuli Venezia Giulia or in the Veneto region (northeastern Italy). None of the participants had prior cancer diagnosis or received previous cancer treatment. Pathological records were centrally reviewed by a pathologist to collect information on PCa characteristics at diagnosis, including Gleason score. Three patients with incomplete dietary data were excluded, thus leaving 777 patients [13].

During routine hospitalization for diagnosis or staging, PCa patients were interviewed by trained personnel using a structured questionnaire including information on socio-demographic characteristics, lifestyle habits, and personal medical history. Anthropometric measures were assessed by the interviewer during the interview; according to the definition by the World Health Organization [14], abdominal obesity was defined as waist circumference >102 cm, measured 2 cm above the umbilicus. For those lacking this information (56 men, 7.7%), a linear regression model predicting waist circumference from body mass index (BMI) was used to approximate abdominal obesity. In the present study cohort, the BMI value predicting abdominal obesity was >27.7 kg/m^2^ [15].

The habitual diet during the two years prior to cancer diagnosis was assessed through a validated and reproducible food-frequency questionnaire (FFQ) [13], including 78 foods, beverages, or recipes structured into seven sections. Participants were asked to indicate the average weekly frequency of consumption of each dietary item, reporting variation in seasonal consumption of fruit and vegetables. The serving size was defined in “natural” units—e.g., one egg (average weight: 65 g), one apple (average weight: 200 g)—or as an average serving in the Italian diet (e.g., 80 g of pasta, 150 g of tomatoes). Intakes lower than once a week but at least once a month were coded as 0.5 per week. Total energy and nutrient intakes were computed using the Italian food composition database [16]. 

Adherence to the Mediterranean diet was investigated using the Mediterranean Diet Score (MDS). This is an a priori score developed using nine dietary indicators [17]: high consumption of cereals, fruit, vegetables, legumes, fish, high monounsaturated/saturated fatty acids (MUFA/SFA) ratio, low consumption of dairy products (including milk) and meat, and moderate alcohol consumption. The nine dietary indicators were expressed in grams per week (g/week) and they were derived from the FFQ by summing the products of the weekly consumption of each food by its corresponding serving weight. High or low consumption was defined according to median value for all food parameters, except for alcohol intake; moderate alcohol consumption was defined as 1–3 drinks/day. For each study participant and each diet indicator, a value of 1 was assigned when the subject fulfilled the MDS requirement, 0 otherwise. The MDS was calculated adding up the values for each of the nine components; thus, the score ranged from 0 (representing minimal adherence) to 9 (maximal adherence). 

Occupational and recreational physical activity were assessed in different periods of life (e.g., at 12 years, 15–19 years, 30–39 years, 50–59 years, and before diagnosis). Patients were asked to self-report intensity of activity at work (i.e., “very strenuous”, “strenuous”, “average”, “standing”, or “mainly sitting”) and during leisure time (i.e., <2, 2–4, 5–7, and >7 h per week) separately (Table S1). Overall physical activity was defined as “low”, “moderate” or “high” by combining occupational and recreational physical activities (Table S1). In the present analysis, physical activity prior to diagnosis was considered as the main exposure. 

The vital status, the date, and the underlying cause of death (i.e., the condition that led to death) were ascertained up to 31 December 2017 through a record-linkage procedure with the population-based regional cancer registries of Friuli Venezia Giulia and Veneto regions [12]. Person-time at risk was computed as the time elapsed from the date of PCa diagnosis to the date of death, to end of follow-up, or to 31 December 2017 (i.e., censored data), whichever came first. To limit the bias of modification of physical activity with increasing age, the follow-up for the present analysis was truncated at 10 years.

An a priori power analysis was conducted to estimate the minimum detectable effect size, given the available number of 780 patients and α = 0.05. Splitting the study population into two equal groups (i.e., exposed and unexposed), the study had a power of 80% to detect an HR ≤ 0.79 when the event rate was 30% in the unexposed group (e.g., all-cause death), HR ≤ 0.78 when the event rate was 20% (e.g., non-PCa death), and HR ≤ 0.72 when event rate was 10% (e.g., PCa death).

Survival analysis was conducted separately for overall survival and cause-specific survival. The overall survival probabilities of PCa patients according to MDS and physical activity were estimated through the Kaplan-Meier method and survival differences were tested through the log-rank test [18]. Hazard ratios (HRs) of all-cause death and corresponding 95% confidence intervals (CIs) were estimated using Cox proportional hazards models [18]. To account for competing risks, cause-specific mortality was evaluated through cumulative incidence [19] and differences according to strata were tested through Gray’s test [19]. HRs were estimated through the Cox model, accounting for competitive risk according to the Fine–Gray model [19]. The proportional hazards assumption was assessed through the Schoenfeld residuals and including interactions with follow-up time [18]. HRs were adjusted for area of residence, year of cancer diagnosis (continuous), age at diagnosis (continuous), education (<7, 7–11, ≥12 years), Gleason score (2–6, 7, 8–10, unknown), smoking habits (never, former, current), abdominal obesity (no, yes), and total energy intake (kJ/day). Interaction between MDS and physical activity was tested through the estimation of relative excess risk due to interaction and the synergic index [20]. Statistical significance was claimed for *p* < 0.05 (two-tailed).

## 3. Results

At PCa diagnosis, 383 (49.3%) patients reported high adherence to the Mediterranean diet (MDS = 5–9); MDS correlated positively with education and inversely with current smoking (Table 1). High physical activity was reported by 346 men (44.5%); it correlated with lower education and higher total energy intake. Patients reporting higher MDS also reported lower intakes of animal fat, animal proteins, and saturated fatty acids together with higher intakes of unsaturated fatty acids, dietary fibre, α- and β-carotene, compared to patients reporting lower MDS (Table S2).

**Figure 1:**
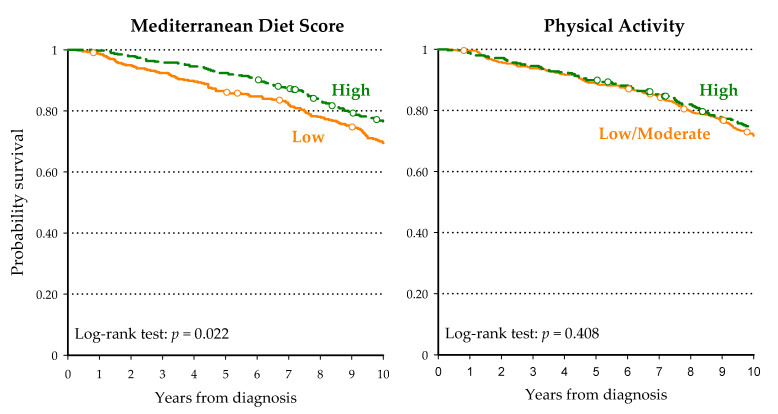
Kaplan–Meier estimates of survival in 777 men diagnosed with prostate cancer, according to Mediterranean Diet Score and physical activity.

During the 10-year follow-up, 208 patients (26.8%) died, with a median time to death of 6.3 years. PCa was the leading cause of death in 75 patients (36.1%), and death was attributed to other causes in 133 patients (63.9%). Among the latter, 66 patients died from a second cancer, 37 from cardiovascular diseases, and 30 from miscellaneous conditions or injuries. Patients highly adherent to the Mediterranean diet reported a better overall survival than those who had low adherence (*p* < 0.01; Figure 1), with a 10-year survival probability of 76.5% and 65.5% for high and low MDS adherence, respectively (Table 2). The advantage in overall survival in men with high MDS compared to low MDS was confirmed by multivariate analysis (Table 2), with HR = 0.74 (95% CI: 0.56–0.99). Survival curves according to physical activity level at the time of diagnosis were largely overlapping, and no significant difference was observed (*p* = 0.408; Figure 1). However, after accounting for potential confounders, the multivariate analysis found a 21% non-significant reduction (95% CI: 0.59–1.07) for men with a high physical activity level before diagnosis. No significant association emerged for physical activity in other periods of life, i.e., at ages 15–19 years (HR = 1.06; 95% CI: 0.76–1.49), 30–39 years (HR = 0.91; 95% CI: 0.67–1.23) and 50–59 years (HR = 0.88; 95% CI: 0.65–1.20; data not shown). Interestingly, physically active men who were highly adherent to the Mediterranean diet reported a lower risk of all-cause death (HR = 0.58; 95% CI: 0.38–0.90) compared to those with low MDS and low/moderate physical activity (Table 2). However, no significant additive or synergic effect was observed (*p*_interaction_ = 0.247).

**Figure 2:**
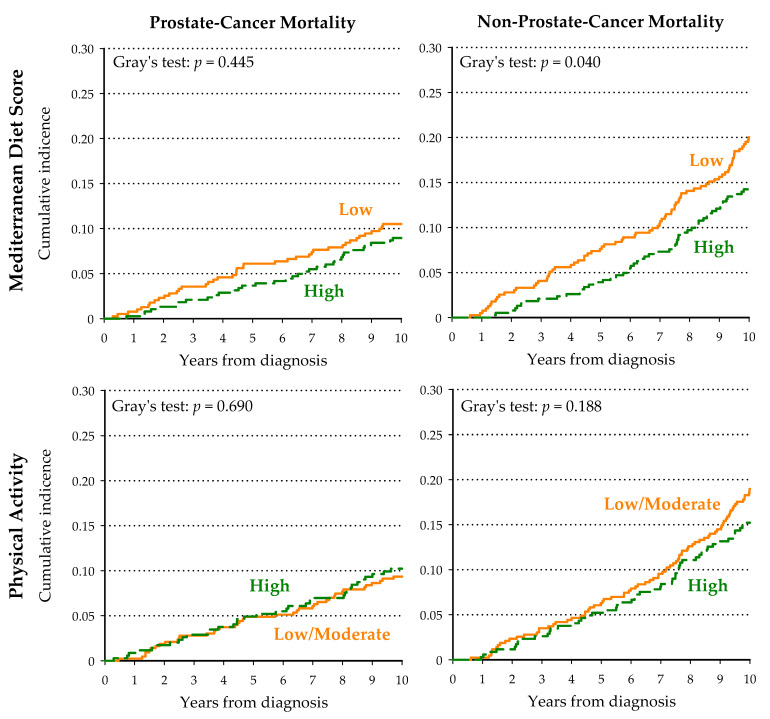
Cumulative incidence of prostate cancer and non-prostate cancer death among 777 men diagnosed with prostate cancer, according to Mediterranean Diet Score and physical activity.

Men with higher adherence to the Mediterranean diet experienced significantly lower cumulative incidence of non-PCa specific death (*p* = 0.040; Figure 2), but not PCa specific death (*p* = 0.445). No significant difference in both PCa specific and non-PCa specific mortality was found according to physical activity level (Figure 2). In a multivariate model, neither high MDS nor high physical activity alone were significantly associated with PCa-specific mortality, but HRs for non-PCa mortality were of borderline significance (Table 3). Notably, the risk of death due to non-PCa related causes was almost halved (HR = 0.51; 95% CI: 0.29–0.91) in physically active men highly adherent to the Mediterranean diet and compared to those scarcely adherent to the Mediterranean diet and with low/moderate physical activity level (Table 3).

## 4. Discussion

The results of the present study support a beneficial effect of pre-diagnostic adherence to the Mediterranean diet and physical activity in reducing the risk of death in men with PCa. Notably, the risk of death after PCa diagnosis, in particular for non-PCa related causes, was almost halved in men adherent to the Mediterranean diet who were also physically active at the time of cancer diagnosis.

Adherence to the Mediterranean diet has been associated with reduced incidence of several health outcomes [21], including overall and cancer mortality [5,21]. Nonetheless, very few studies have been conducted on survival in cancer patients. Patients adherent to the Mediterranean diet reported better prognosis after colorectal cancer [22] and post-menopausal breast cancer [23]. Only one study focused on prostate cancer [6], reporting a mortality risk reduction similar to that found in our study. Conversely, the present study did not find an association between Mediterranean diet and PCa-specific mortality. No study investigated Mediterranean diet in relation to this outcome, but indirect evidence emerged from interventional studies on specific nutritional supplements which did not find an association with PCa progression or mortality [24]. 

Physical inactivity is a major risk factor for mortality, mainly due to increased cardiovascular and cancer mortality [25,26]; however, the advantage in overall survival compared to inactive people is generally appreciable for vigorous rather than for moderate physical activity [25,26]. Cohort studies consistently reported approximately 40% reduction in PCa mortality in physically active men, despite the diverse methods for assessing physical activity [9,10]. Interestingly, a similar beneficial effect of physical activity was reported for patients with breast and colorectal cancer [27]. Consistently, physical activity was also associated with reduced overall mortality [7,8,9,10].

The Mediterranean diet and physical activity share some common mechanisms which may explain the reduction in mortality in PCa patients. These pathways involve insulin, insulin-like growth factors (IGF), and inflammation. Barnard and colleagues [28,29] reported that the combination of low-fat, high-fibre diets and intense daily physical activity lowered serum insulin levels and insulin resistance while increasing IGF binding protein 1. Although some reduction of growth factors levels was appreciable with physical activity alone, the effect was magnified by the introduction of the low-fat, high-fibre diet in the study by Barnard et al. [28]. Interestingly, when serum from men enrolled in the dietary study by Barnard and colleagues was used to grow PCa epithelial cells, the authors reported that the low-fat, high-fibre diet and intense daily physical activity induced a reduction in growth of PCa epithelial cells and androgen-sensitive PCa cells [29], as well as their apoptosis [28].

Data on modifications of diet, physical activity, and weight after PCa diagnosis were not assessed in the present study. However, it is unlikely that changes in dietary habits had occurred in the Italian male population at that time and at their age since the general population had not been made aware of any putative association between diet and cancer survival, and no dietary guidelines existed for patients with PCa. Furthermore, studies investigating dietary modifications after PCa diagnosis did not report substantial changes in the mean intake of foods [30]. Regarding physical activity, it is plausible that the intensity may be lower with increasing age, thus producing misclassification, even if physically active men at cancer diagnosis have a greater chance to remain active thereafter. To limit this possible source of bias, the analysis was truncated at 10 years after diagnosis. Nonetheless, this misclassification bias was likely to reduce the estimated effect of physical activity rather than enhance it, so that our risk estimates were conservative. It is worth noting that results from the CPS-II Nutrition Cohort [9] showed a similar beneficial effect of pre-diagnostic and post-diagnostic recreational physical activity in men with Gleason 2–7 PCa. However, modification in physical activity level after PCa may vary according to disease severity, since patients with advanced cancer may be cachexic and unable to maintain the physical activity level assessed at the time of diagnosis. Finally, the lack of specific information on type and intensity of physical activity may have produced information bias. The lack of some relevant clinical information (e.g., PSA at diagnosis, staging) and treatments type and completion should also be recognized among the study limitations. However, our model included Gleason score as a covariate; although it is not a perfect prognostic factor, it is quite a reliable indicator of disease severity and an acceptable predictor of prostate cancer mortality in observational studies [31]. Furthermore, men highly adherent to the Mediterranean diet reported higher education, thus influencing their need for treatment and affecting their survival differently than men with lower adherence to the Mediterranean diet. Selection bias may also have occurred, but it was minimized in the original study by including all newly diagnosed PCa patients consecutively admitted to the major local hospitals in the study areas with a refusal rate of approximately 4%. Patients lost at follow-up were below 3%. Therefore, our study population was representative of men with PCa in the study areas [32]. Finally, a post hoc power analysis revealed low power (i.e., a type II error probability of about 30%) for PCa-specific survival, which calls for caution in interpreting the results.

## 5. Conclusions

Consistently with many prior studies, albeit few in cancer survivors, findings of the present study support the beneficial impact of pre-diagnostic adherence to the Mediterranean diet and physical activity on overall survival after prostate cancer diagnosis, mainly due to lower non-PCa specific mortality. Although we found no suggestion that the Mediterranean diet was associated with reduced cancer-specific mortality, these findings support trials to test whether this diet can reduce non-PCa-specific mortality, which accounts for about 82% of deaths in men with PCa [4].

## Figures and Tables

**Table 1:** Distribution of 777 men diagnosed with prostate cancer, according to baseline characteristics, Mediterranean Diet Score (MDS) and physical activity. Italy, 1995–2002.

	MDS				Physical Activity			
	Low (0–4)		High (5–9)		Low/Moderate		High	
	*n*	(%)	*n*	(%)	*n*	(%)	*n*	(%)
Age (years)								
<65	158	(40.1)	156	(40.7)	178	(41.3)	136	(39.3)
≥65	236	(59.9)	227	(59.3)	253	(58.7)	210	(60.7)
	*p* = 0.858				*p* = 0.574			
Education (years)								
<7	230	(58.4)	166	(43.3)	162	(37.5)	234	(67.6)
7 to 11	94	(23.9)	133	(34.7)	157	(36.3)	71	(20.5)
≥12	70	(17.8)	84	(21.9)	113	(26.2)	41	(11.9)
	*p* < 0.001				*p* < 0.001			
Gleason score								
2–6	195	(49.5)	200	(52.2)	232	(53.8)	163	(47.1)
7	83	(21.1)	81	(21.2)	92	(21.3)	72	(20.8)
8–10	73	(18.5)	47	(12.3)	59	(13.6)	61	(17.6)
Unknown	43	(10.9)	55	(14.4)	48	(11.1)	50	(14.5)
	*p* = 0.071				*p* = 0.149			
Tobacco smoking								
Never	121	(30.7)	104	(27.2)	125	(29.0)	100	(28.9)
Former	185	(47.0)	214	(55.9)	219	(50.8)	180	(52.0)
Current	88	(22.3)	65	(17.0)	87	(20.2)	66	(19.1)
	*p* = 0.035				*p* = 0.916			
Central obesity ^a^								
No	256	(65.0)	254	(66.3)	281	(65.2)	229	(66.2)
Yes	138	(35.0)	129	(33.7)	150	(34.8)	117	(33.8)
	*p* = 0.693				*p* = 0.773			
Total energy intake (kJ)								
<9811	133	(33.8)	125	(32.6)	178	(41.3)	80	(23.1)
9811 to <12,502	130	(33.0)	129	(33.7)	151	(35.0)	108	(31.2)
≥12,502	131	(33.3)	129	(33.7)	102	(23.7)	158	(45.7)
	*p* = 0.946				*p* < 0.001			

^a^ Defined as waist circumference >102 cm (or BMI >27.7 kg/m^2^ when information on waist was missing).

**Table 2:** Kaplan–Meier estimates of survival and hazard ratios (HR) of death and corresponding 95% confidence intervals (CI), among 777 men diagnosed with prostate cancer, according to Mediterranean Diet Score and physical activity. Italy, 1995–2002.

Score	Patients	Deaths		Survival Probabilities			HR (95% CI) ^a^
		n	(%)	5 Years	10 Years	15 Years	
**Mediterranean Diet Score**							
Low (0–4)	394	180	(45.7)	86.3%	69.5%	50.7%	Reference
High (5–9)	383	151	(39.4)	92.4%	76.5%	57.3%	0.80 (0.60–1.00)
**Physical activity**							
Low/Moderate	389	183	(47.0)	87.6%	69.3%	49.5%	Reference
High	388	148	(38.1)	91.0%	76.6%	58.5%	0.79 (0.63–0.98)
**Mediterranean Diet Score and Physical activity**							
Low and Low/Moderate	197	66	(33.5)	89.8%	84.2%	66.2%	Reference
Low and High	197	53	(26.9)	94.9%	88.3%	72.8%	0.70 (0.48 to 1.02)
High and Low/Moderate	234	55	(23.5)	97.0%	92.7%	76.3%	0.66 (0.46 to 0.95)
High and High	149	34	(22.8)	94.0%	91.3%	76.9%	0.58 (0.38 to 0.90)

^a^ Estimated using Cox proportional hazard model adjusted for area of residence at diagnosis, calendar period of cancer diagnosis, age at diagnosis, years of education, Gleason score, abdominal obesity, tobacco smoking, and total energy intake.

**Table 3:** Hazard ratios (HR) ^a^ for prostate cancer (PCa) and non-PCa mortality, with corresponding 95% confidence intervals (CI), among 777 men diagnosed with prostate cancer, according to adherence to Mediterranean Diet Score and physical activity. Italy, 1995–2002.

Score	Patients	PCa Mortality			Non-PCa Mortality		
		Events		HR (95% CI)	Events		HR (95% CI)
		n	(%)		n	(%)	
**Mediterranean Diet Score**							
Low (0–4)	394	41	(10.4)	Reference	78	(19.8)	Reference
High (5–9)	383	34	(8.9)	0.83 (0.53 to 1.31)	55	(14.4)	0.73 (0.51 to 1.05)
**Physical activity**							
Low/Moderate	427	40	(9.4)	Reference	81	(19.0)	Reference
High	346	35	(10.1)	0.95 (0.57 to 1.59)	52	(15.0)	0.72 (0.49 to 1.08)
**Mediterranean Diet Score and Physical activity**							
Low and Low/Moderate	197	21	(10.7)	Reference	45	(22.8)	Reference
Low and High	197	19	(8.1)	0.85 (0.44 to 1.65)	36	(15.4)	0.67 (0.41 to 1.10)
High and Low/Moderate	234	20	(10.2)	0.75 (0.41 to 1.38)	33	(16.8)	0.68 (0.43 to 1.07)
High and High	149	15	(10.0)	0.79 (0.40 to 1.59)	19	(12.8)	0.51 (0.29 to 0.91)

^a^ Estimated using Cox proportional hazard model adjusted for age at diagnosis, years of education, Gleason score, abdominal obesity, smoking habits, and total energy intake. Competitive risks were accounted according to Fine-Gray model.

## Data Availability

Data is available for research purpose upon reasonable request to the corresponding author.

## Supplementary Materials

The following are available online at https://www.mdpi.com/2072-6643/13/1/243/s1, Table S1: Definition of physical activity according to levels of recreational and occupational physical activity; Table S2: Nutrients median values and interquartile range (Q1-Q3), among 777 men diagnosed with prostate cancer, according to according to Mediterranean Diet Score (MDS) and physical activity. Italy, 1995–2002
