# Supplementary material for: Adherence to Mediterranean Diet, Physical Activity and Survival after Prostate Cancer Diagnosis
Source: Nutrients. 2021 Jan 16;13(1):243. doi: 10.3390/nu13010243 (PMC7829941; doi:10.3390/nu13010243)
Supplement: Supplementary file 1 [file nutrients-13-00243-s001.pdf]

Table S1. Definition of physical activity according to levels of recreational and occupational physical activity.

Overall physical activity	Recreational physical activity	Occupational physical activity
**High**	>7 hours/week	Any
	Any	Very strenuous (e.g. bricklayer, carpenter) or strenuous (e.g. farmer, metal worker)
	5-7 hours/week	Average (e.g. cook, waiter)
**Moderate**	5-7 hours/week	Standing (e.g. teacher, salesman/saleswoman) or Mainly sitting (e.g. clerk)
	2-4 hours/week	Average (e.g. cook, waiter)
**Low**	≤4 hours/week	Standing (e.g. teacher, salesman/saleswoman) or Mainly sitting (e.g. clerk)

Table S2. Nutrients median values and interquartile range (Q1-Q3), among 777 men diagnosed with prostate cancer, according to according to Mediterranean Diet Score (MDS) and physical activity. Italy, 1995-2002

**Daily nutrient intake**	**Low MDS**		**High MDS**		**Kruskal-Wallis test**
	**Median**	**(Q1-Q3)**	**Median**	**(Q1-Q3)**	
Total proteins (g)	98.4	(83.3-115.8)	90.2	(74.9-110.0)	P<0.001
Vegetal proteins (g)	30.6	(25.4-35.4)	35.2	(29.2-42.4)	P<0.001
Animal proteins (g)	68.5	(57.4-82.1)	54.3	(43.8-67.9)	P<0.001
Total fats (g)	90.7	(73.1-117.1)	88.2	(71.2-113.9)	P=0.180
Vegetal fats (g)	40.5	(28.7-52.3)	46.5	(33.1-61.8)	P<0.001
Animal fats (g)	50.3	(39.7-63.1)	39.4	(31.3-51.8)	P<0.001
Total carbohydrates (g)	279.9	(229.1-334.0)	307.4	(248.9-372.8)	P<0.001
Sugars (g)	92.8	(68.6-116.0)	98.0	(77.4-122.8)	P=0.014
Starch (g)	185.7	(153.8-222.8)	202.1	(161.5-257.3)	P<0.001
Fibre (g)	20.7	(16.4-24.2)	25.9	(21.6-31.5)	P<0.001
Saturated fatty acids (g)	31.0	(24.8-39.0)	27.2	(21.4-36.7)	P<0.001
MUFA (g)	39.4	(30.4-50.3)	42.6	(32.5-55.5)	P=0.008
PUFA (g)	13.8	(10.5-19.5)	11.7	(9.1-15.8)	P<0.001
Cholesterol (mg)	358	(288-455)	301	(234-380)	P<0.001
Calcium (g)	1.18	(0.91-1.46)	0.96	(0.80-1.29)	P<0.001
Sodium (g)	2.43	(1.99-3.01)	2.41	(1.90-3.05)	P=0.550
Potassium (g)	3.90	(3.29-4.49)	4.03	(3.45-4.75)	P=0.007
Iron (mg)	16.8	(14.2-20.1)	16.4	(13.2-19.3)	P=0.058
Thiamin (mg)	0.84	(0.71-0.98)	0.87	(0.74-1.05)	P=0.054
Riboflavin (mg)	1.64	(1.37-2.07)	1.55	(1.26-1.90)	P=0.001
Vitamin C (mg)	95.5	(67.6-127.1)	131.8	(102.1-174.9)	P<0.001
Vitamin B6 (mg)	1.92	(1.64-2.26)	1.94	(1.65-2.31)	P=0.386
Folate (μg)	241	(197-287)	274	(231-321)	P<0.001
Niacin (mg)	18.0	(14.9-20.6)	17.4	(14.7-20.7)	P=0.568
α-Carotene (μg)	383	(185-692)	675	(363-1125)	P<0.001
β-Carotene (mg)	3.60	(2.74-4.81)	4.93	(4.01-6.21)	P<0.001
Vitamin D (μg)	3.07	(2.33-3.99)	3.07	(2.23-3.98)	P=0.811
Vitamin E (mg)	13.5	(10.9-17.4)	15.6	(12.6-20.2)	P<0.001
Alcohol (g)	52.4	(28.1-77.8)	29.0	(13.8-53.9)	P<0.001
Water (l)	1.72	(1.44-2.08)	1.70	(1.39-2.01)	P=0.429
Total energy (kJ)	6362	(5374-7559)	6194	(5047-7431)	P=0.144
